# The future of carbon-neutrality science and technology from an industrial transformation perspective: an interview with Hou-Liang Dai

**DOI:** 10.1093/nsr/nwac295

**Published:** 2022-12-29

**Authors:** By Hai-Xia Huang

**Affiliations:** Science and Technology Department of the China National Petroleum Corporation

## Abstract

Since China announced that it will strive to achieve its carbon peak by 2030 and carbon neutrality by 2060, it has determined to take steps, which will establish low carbon development and a transition to carbon neutrality, as part of its long-term strategy for sustainable development and prosperity. This will be both a huge challenge and an opportunity. To achieve the dual-carbon goals, the whole society must undergo a broad and profound systemic transformation, involving changes in energy, technology, economy, industry and lifestyle, etc. Carbon-neutrality science and technology should be the foundation of the transformation.

Now, dual-carbon goals have become the focus, not just of scientific and technological circles [1], but of all society. Some Chinese scientists have put forward the concept of green carbon science (GCS) to promote rational and systematic scientific thinking on carbon science and sustainable development [2,3]. Additionally, hundreds of scientists and senior experts have participated in formulating the action plan and guiding the outline of basic research on the dual-carbon strategy and pathways, helping the country to formulate a timetable, roadmap and implementation plan for achieving carbon-peaking and carbon-neutralization goals in industries [4,5]. Among them, Hou-Liang Dai, an academician of the Chinese Academy of Engineering (CAE), took part in a major consulting project of the CAE. He is famous for the development and application of highly efficient and environmentally friendly aromatic hydrocarbon technology and also a leader in high-quality development of the petrochemical industry [6]. He has been working in the petroleum and chemical industry for almost 40 years. Currently, he is the chairman of the China National Petroleum Corporation (CNPC).

NSR spoke to Hou-Liang Dai on the future of carbon-neutrality science and technology.

***NSR*:** As we know, China has proposed ‘2060’ carbon-peak and carbon-neutrality goals. How do you think China’s energy system will change in the future? In the process of green and low-carbon development of the energy system, how do you think China’s traditional fossil energy industry will reform?

***Dai*:** China has established a ‘1 + N’ policy framework for dual-carbon goals to promote green and low-carbon development of the energy system. It is expected that the proportion of non-fossil energy consumption will reach 25% and surpass 80% by 2030 and 2060, respectively, meaning that there will be fundamental changes in China's energy system. According to the national dual-carbon strategy, China's energy mix will change from the current coal-dominated structure supplemented by oil, natural gas and non-fossil energy, to a non-fossil-energy-dominated structure supplemented by coal, oil and natural gas in the future. Wind, solar, biomass and other renewable energy and nuclear energy will gradually become the focus for the development of the new energy system.

In industry, traditional fossil energy will gradually exit in an orderly manner and new energy will take its place. However, the transformation of China's energy mix should be based on the national resource endowment and optimized safely and steadily. In any case, the bottom line is to ensure national energy security and economic development. We should adhere to the principle of establishment before breakthrough, and strengthen the coordinated development and integration of traditional fossil energy industries with new energy.

**Figure 1:**
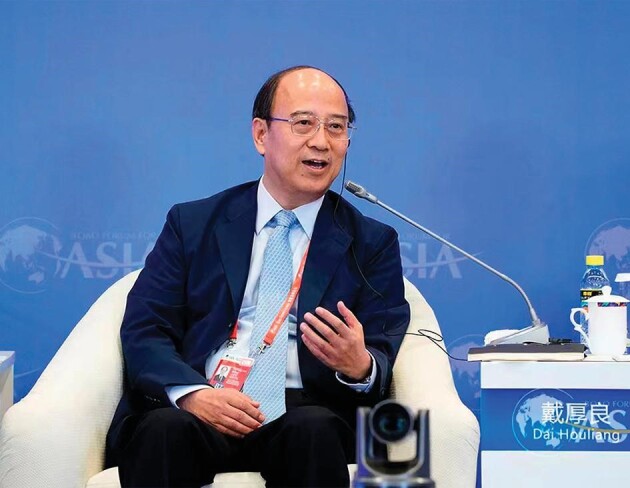
Hou-Liang Dai, Chairman of the China National Petroleum Corporation, also an academician of the Chinese Academy of Engineering (*courtesy of ‘Boao Forum for Asia’*).

A lot can be done with regard to traditional fossil energy companies achieving green and low-carbon development. On one hand, they should strengthen the clean and efficient development and utilization of fossil energy, promote the transformation of non-fossil energy from supplementary energy to

We should adhere to the principle of establishment before breakthrough, and strengthen the coordinated development and integration of traditional fossil energy industries with new energy.

—Hou-Liang Dai

alternative energy, and actively build digital and intelligent energy integrated systems to further improve the efficiency of energy allocation and utilization. On the other hand, since fossil energy will be less used as fuel in the future, R&D for new high-value-added chemicals and synthetic chemicals becomes necessary. Enterprises should be encouraged to shift their activities from type of fuel and raw materials to raw-material-oriented type, by strengthening R&D systems for high-end raw materials from fossil resources.

***NSR*:** How does the industry treat CO_2_ emissions at present? In your opinion, is it possible to realize large-scale CO_2_ recycling and utilization in the future?

***Dai*:** At present, carbon capture, utilization and storage technology (CCS/CCUS) is recognized as the most promising and large-scale key technology for reducing CO_2_ emissions from industry. CCS, first proposed in the 1970s, refers to capturing CO_2_ from carbon-intensive industries, compressing it to a supercritical state and injecting it into underground formations, such as deep salt beds or depleted oil and gas reservoirs, for permanent storage. A preliminary estimate for the potential space for geological sequestration in China is ∼1.2–4.1 trillion tons. However, CCS technology faces the problems of relative high energy consumption and high operation cost for pipeline transportation and geological storage. Besides, due to storage limitations, the annual storage capacity of CCS facilities around the world has only reached one thousandth of annual CO_2_ emissions. Subsequently, in the last two decades, the CCS concept has evolved into CCUS, which is more practical and economically feasible than CCS, because CO_2_ utilization units may produce economic benefits via CO_2_ flooding to enhance oil and gas recovery (CO_2_-EOR), or via conversion of CO_2_ to high-value products through various chemical and biological processes, such as chemical conversion of CO_2_ into fuels and chemicals, CO_2_ mineralization, and microalgae cultivation. In China, CNPC Company was the first to carry out the industrialization exploration of the CCUS-EOR project and has carried out 11 CCUS pilot tests in 10 oil and gas fields with accumulated buried carbon dioxide exceeding 4.5 million tons. Also, China's first million-ton-level CCUS-EOR project has been fully constructed since 2022. It is expected that, more and more, such CCUS projects will be built in China in the near future.

On the other hand, some chemical utilizations of CO_2_ for e.g. hydrogenation to methanol, reforming with CH_4_ to produce syngas, and the synthesis of acetic acid and organic fine chemical products, are under research and development. Some are close to breakthrough. It is expected that these technologies will become commercially available in the near future, after overcoming the biggest challenge of economic viability.

In the future, integration of green electricity, ‘green hydrogen’ and CCUS technologies may bring a revolution to fossil-energy-based industrial processes such as steel production, cement factories, coal power plants and the coal chemical industry. I believe that in the future it will be possible to achieve CO_2_ emissions reduction on a large scale or even near-zero carbon emissions. In general, according to International Energy Agency (IEA) estimates, CCUS will contribute 8% of the total cumulative CO_2_ emissions reduction from now to 2060.

***NSR*:** What is your opinion on the development of hydrogen energy?

***Dai*:** As a renewable, clean and efficient secondary energy, hydrogen will be an important energy source or medium for realizing the energy transition or carbon-neutrality goals in the future. Currently, 41 countries around the world have issued the ‘National Hydrogen Energy Development Strategy’, and development of hydrogen energy has become a strategic choice for many countries trying to promote the energy transition. It is likely that hydrogen energy will be a ‘bridge’ connecting renewable energy with the electrical power system, fossil energy industry, transportation industry and other fields in future societies, playing an important role in achieving green and low-carbon goals.

Hydrogen can be produced from fossil energy, water electrolysis, industrial by-products and renewable energy. Among them, green hydrogen, which is produced using electricity from renewable resources, could be the key to curbing our carbon footprint in the future. However, due to its low electrolysis efficiency and high production cost, less than 1% of total annual hydrogen production is ‘green’ today. So, as we can see, hydrogen energy is still in the early stage of development and faces many challenges at present. Technical breakthroughs are expected on advanced solid oxide electrolyzers with high electrical efficiency and low operation costs for hydrogen production. Also, there is a general lack of hydrogen infrastructure, including efficient transportation and storage of hydrogen. In addition, the application scenarios of hydrogen energy are relatively simple. At present, hydrogen is mainly used as a chemical raw material for the production of methanol, synthetic ammonia and various chemical products, while its use in fuel cells is <0.1%. The current practical choice is ‘blue hydrogen’, which is produced in much the same manner as ‘gray hydrogen’, but the CO_2_ emissions

As a renewable, clean and efficient secondary energy, hydrogen will be an important energy source or medium for realizing the energy transition or carbon-neutrality goals in the future .

—Hou-Liang Dai

Green carbon science is at the center of the interconnected triad of sustainable development, fossil energy and CO_2_, playing a supporting and coordinating role as a scientific foundation.

—Hou-Liang Dai

are captured and stored underground (the CCS process). It is estimated that it will still take at least 10 years to reduce the cost of green hydrogen enough so that it approaches the cost of gray hydrogen. China is vigorously promoting the development of the hydrogen energy industry, including the construction of thousands of oil-gas-electricity-hydrogen combined fueling stations and huge hydrogen-energy-industry clusters.

***NSR*:** What is your view on the future of biomass energy and biomass conversion technology?

***Dai*:** Biomass resources come from agricultural waste, wood and forest waste, municipal organic waste, algal biomass, energy crops and so on. Biomass has been widely used in many fields such as industry, agriculture, transportation and life, through power generation, heating and gas supply. Biomass energy is energy in the form of biodiesel, bioethanol, biogas etc. generated from biomass through chemical conversion. As the only internationally recognized zero-carbon fuel among renewable energy sources, biomass energy will be an important part of global low-carbon-energy systems in the future. Biofuels are a direct and effective means to reduce the carbon emission levels of the aviation industry and realize the replacement or supplement of fossil energy in the future. Sustainable utilization of biomass resources and organic waste will be conducive to optimizing the energy structure and achieving the dual-carbon goals.

The potential biomass energy in China is equivalent to ∼460 million tons of standard coal every year. However, current utilization is only 60 million tons of standard coal. Biomass energy development faces many challenges in China, such as insufficient supply of stable and cheap raw materials, lack of fiscal and tax policy support, inadequate technological innovation, immature industrialization technology, small industrialization scale and incomplete industrial chain.

So, high-quality development of the biomass energy industry requires not only adequate scientific and technological innovation, but also strong policy support. On the one hand, relevant innovations are needed in biomass conversion technology and key equipment industrialization. Biomass energy should not be mainly used for power generation. It needs to develop high added value via diverse and efficient utilization. On the other hand, a flourishing biomass energy industry needs to construct a complete production and consumption system, and a complete industry chain as support. Governments should create favorable conditions and a policy environment to promote the development of the biomass energy industry, by, for example, improving the market access of biomass energy products, establishing sufficient financial subsidy policies and strengthening financial support for biomass energy enterprises.

Now, the development of the biomass energy industry in China has ushered in an important window of opportunity under China's strong green commitment. Relevant enterprises should seize their chance and make great progress, I suggest.

***NSR*:** As you know, in recent years some Chinese scientists have put forward the new concept of GCS. What is your comment on it? And what major revolutionary breakthroughs do you expect to be achieved through GCS research?

***Dai*:** Yes, I firstly learned the concept of GCS from Ming-Yuan He *et al*. [3] several years ago. As far as I know, similar to the role of green chemistry, GCS is at the center of the interconnected triad of sustainable development, fossil energy and CO_2_, playing a supporting and coordinating role as a scientific foundation. This concept provides rational and systematic scientific thinking on carbon science, including efficient and green utilization of carbon resources, partial replacement of fossil energy by new energy, reduction of carbon emissions and implementation of carbon recycling, which are of great importance for the sustainable development of our society. In order to achieve the above goals, including carbon neutrality, I believe that it is necessary to carry out extensive basic research on GCS to bring about technological breakthroughs with regard to those aspects.

At present, scientific GCS research has been carried out and is in progress in many fields, such as low-carbon utilization of fossil energy, development of renewable energy and hydrogen energy, and exploration of biomass and carbon dioxide conversion to fuels and chemicals. In the future, once the technologies represented by clean and efficient utilization of coal, *in-situ* conversion and extraction of low-and-medium-maturity shale oil, large-scale effective CCUS and controllable nuclear fusion have achieved their breakthroughs, it is expected that carbon neutrality in the energy sector in China will be achieved ahead of schedule.

## Notes

Hai-Xia Huang is a professor at the Science and Technology Department of the China National Petroleum Corporation.
